# Fiber supplementation protects from antibiotic-induced gut microbiome dysbiosis by modulating gut redox potential

**DOI:** 10.1038/s41467-023-40553-x

**Published:** 2023-08-24

**Authors:** Swathi Penumutchu, Benjamin J. Korry, Katharine Hewlett, Peter Belenky

**Affiliations:** 1 Department of Molecular Microbiology and Immunology, Brown University, Providence, RI 02912 USA; 2 Department of Pathology and Laboratory Medicine, University of Pennsylvania, Philadelphia, PA 19104 USA

**Keywords:** Microbiome, Bacterial host response

## Abstract

Antibiotic-induced gut dysbiosis (AID) is a frequent and serious side effect of antibiotic use and mitigating this dysbiosis is a critical therapeutic target. We propose that the host diet can modulate the chemical environment of the gut resulting in changes to the structure and function of the microbiome during antibiotic treatment. Gut dysbiosis is typically characterized by increases in aerobic respiratory bacterial metabolism, redox potential, and abundance of Proteobacteria. In this study, we explore dietary fiber supplements as potential modulators of the chemical environment in the gut to reduce this pattern of dysbiosis. Using defined-diets and whole-genome sequencing of female murine microbiomes during diet modulation and antibiotic treatment, we find that fiber prebiotics significantly reduced the impact of antibiotic treatment on microbiome composition and function. We observe reduced abundance of aerobic bacteria as well as metabolic pathways associated with oxidative metabolism. These metatranscriptomic results are corroborated by chemical measurements of eH and pH suggesting that fiber dampens the dysbiotic effects of antibiotics. This work indicates that fiber may act as a potential therapeutic for AID by modulating bacterial metabolism in the gut to prevent an increase in redox potential and protect commensal microbes during antibiotic treatment.

## Abstract (web summary)

Here, the authors show that fiber supplementation protects from antibiotic-induced gut microbiome damage by reducing the abundance of aerobic bacteria as well as metabolic pathways associated with oxidative metabolism.

## Introduction

Antibiotics are a crucial part of modern medicine allowing for defense against infection, but their use often results in collateral damage to the gut microbiome^1–3^. This AID can lead to health complications such as inflammatory bowel disease, aberrant immune function, infection and metabolic disorders^4^. Several studies have explored methods to decrease antibiotic stress to the microbiome using oral drug adsorbents and probiotic supplements^5,6^.

However, these approaches can reduce drug efficacy or increase gut disequilibrium in the case of probiotics^6^. In this work, we use diet to modify the gut chemical environment and investigate how fiber prebiotics can alleviate AID by preventing the increase in gut redox potential seen post-antibiotic treatment^4,7,8^.

The type of carbon source in the diet can determine which electron acceptors reach bacteria in the gut driving specific and predictable biochemical reactions^9–11^. For example, simple carbon sources present in the Western high-sugar diet are quickly absorbed by the host, limiting carbon for microbes in the gut. As these microbes compete for the limited carbon available, they metabolize host-derived carbon from mucosal linings in the intestine^12,13^. This, as a result, increases gut inflammation and changes the structure of the microbiome by selecting for bacteria that thrive in this inflammatory and aerobic environment^14^. This dysbiotic environment can provide electron acceptors such as O_2_, NO_3_, Fe^3+^ and thermodynamically select for metabolic reactions with higher redox potential energy^11,15^. On the other hand dietary fiber selects for microbes that can metabolize complex polysaccharides using fermentative metabolism. Short-chain fatty acids (SCFAs) produced by bacteria through fermentation are metabolized by colonocytes in an oxygen consuming reaction^16–18^. As a result of this anaerobic environment, metabolic reactions with lower redox potential energy are thermodynamically favored, such as fermentation.

A current perspective about bacterial susceptibility to antibiotics suggests that modifying metabolism could protect from antibiotic stress. Several in vitro studies have tested this hypothesis and found that repressing microbial metabolism decreases susceptibility to antibiotics^19–22^. These studies suggest that susceptibility is associated with signatures of metabolic activity such as futile cycle upregulation, ATP turnover, higher membrane potential and increased radical species. Conversely, elevated pH, uncoupling electron transport and decreasing glucose availability have all been shown to suppress microbial metabolism and protect from antibiotics^20,22^. This metabolism-driven mechanism of susceptibility has largely been investigated *in vitro,* however, recent work also suggests that modulating the metabolism of gut bacteria in the host could also impact AID.

Several recent studies have begun to explore the role of host-diet on AID. Diet derived fibers such as Xanthan gum^23^ have been shown to protect from the drop in bacterial diversity seen post-antibiotic treatment. Studies have begun to show that a high-fat, high-sugar Western style diet can exacerbate AID^2,3,24^, and in vitro supplementation with prebiotic fiber can protect gut commensals from antibiotics. These associations are promising but there are significant knowledge gaps in the mechanisms behind diet and antibiotic interactions in vivo. In this study, we use metagenomic and metatranscriptomic sequencing of the gut microbiome to acquire high-resolution data of the bacterial composition and function. We combine this sequencing data with chemical measurements to provide context for enriched metabolic pathways. We found that fiber supplementation reduced AID when given before, during or after antibiotic treatment via a redox driven mechanism.

## Results and discussion

### Fiber protects from AID before, during and after antibiotic treatment

**Fig. 1:**
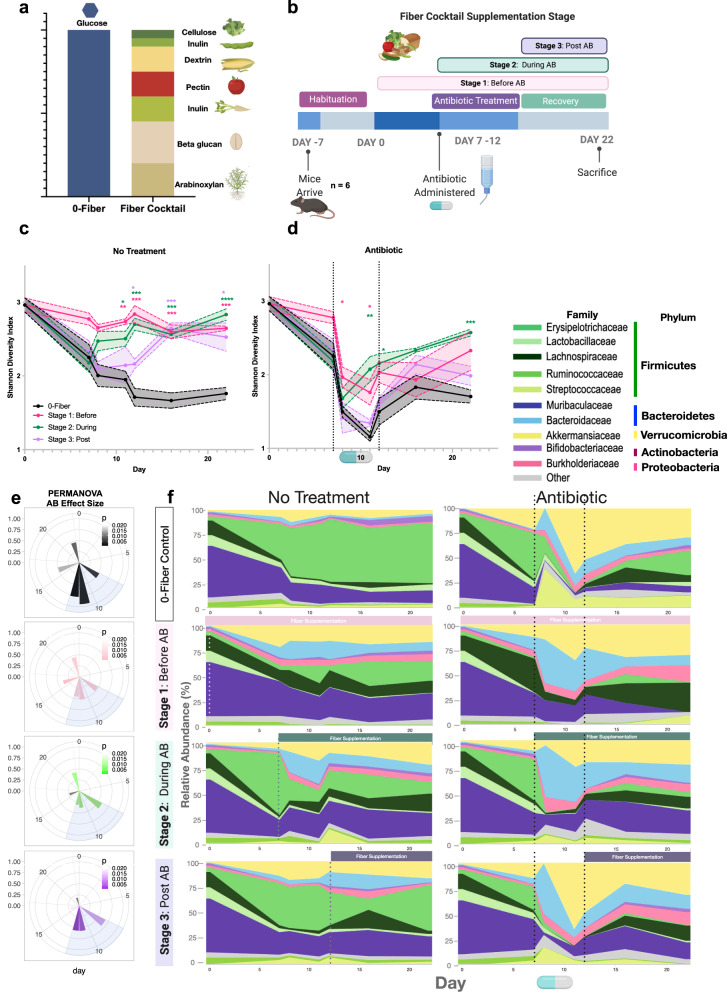
Fiber supplementation alleviates antibiotic-induced dysbiosis before, during, and after antibiotic treatment. Modified versions of the AIN-93G purified rodent diets supplemented with purified fibers were used to modulate carbon source to the gut microbiome. The 0-fiber received no fiber supplement, and 100% glucose was added at a 20% ratio to the diet. The fiber-supplemented mice received a cocktail of 7 purified-plant fibers in the ratios depicted. (**a**) Mouse diet and (**b**) antibiotic intervention schematic. Mean Shannon diversity (*n* = 12 for day 0,7)(*n* = 6 for remaining timepoints) shown for Stage 1 (pink), Stage 2 (green), and Stage 3(purple) are shown with SEM intervals in control (**c**). Colored stars correspond to magnitude of *p* value according to Two-Way Mixed model ANOVA & Dunnett. *p < 0.05; **p < 0.01; ***p < 0.001; ****p < 0.0001. *p* values at day 11: pink – 0.0014, green – 0.0118. *p* values at day 12: pink – 0.0002, green – 0.0004, purple 0.0309. *p* values at day 16: pink – 0.0003, green – 0.0006, purple 0.0003. *p* values at day 22: pink – 0.0001, green – <0.0001, purple 0.0207. Antibiotic treated mice shown in (**d**). *p* values at day 8: pink – 0.0452. *p* values at day 11: pink – 0.0363, green – 0.0060. *p* values at day 12: green 0.0428. *p* values at day 22: green 0.0001. Each stage is compared to 0-fiber unsupplemented diet. Antibiotic effect size calculated with PERMANOVA at all supplementation stages was calculated using Bray–Curtis distance values and the PCoA method. Color intensity represents *p* value (scale displayed). Full results shown in source data (**e**). Relative abundances of bacterial families shown throughout the course of the experiment (**f**).

We used female C57BL/6 mice to test the effects of purified-plant fiber supplementation on AID in mice fed the purified AIN-93G diet (Envigo-Teklad). This diet is prepared at 80% composition, allowing for 20% supplementation of a carbon source. We used glucose as our low-fiber unsupplemented condition, and a cocktail of 7 plant fibers including (cellulose, levan, dextrin, pectin, inulin, beta-glucan, arabinoxylan) (Fig. 1a) for the fiber-supplemented conditions. Glucose was chosen as the no fiber addition to maintain carbohydrate:fat:protein ratios and reduce microbiota accessibility. Fiber-free diets typically contain simple-sugars in place of complex polysaccharides as shown in Desai et al.^25^ and Kamada et al.^26^. Simple-sugars are used because they are very host accessible and are likely to be processed in the small intestine limiting access to microbiota^10^. Additionally, glucose is a common dietary component that may have fewer detrimental effects to the host than other monosaccharides^27^ such as fructose which has been to shown to have kidney toxicity in the short-term^27^.

We used longitudinal 16S rRNA sequencing of feces to assess the optimal stage of fiber supplementation on post-amoxicillin recovery (150 mg/kg *ad libitum* in water) (Fig. 1b). Non-significant differences in weight were observed between groups (Supplementary Fig. 1a). Glucose in the absence of antibiotic was observed to decrease microbial diversity throughout the experiment (Fig. 1c). Fiber supplementation before antibiotic (AB) treatment ([S1]:Before) had a significantly lower (*p* < 0.05) initial reduction in diversity and a more complete recovery compared to the glucose group (Fig. 1d). Similarly, fiber supplementation during antibiotic treatment ([S2]:During) also conferred significant protection in both the treatment (*p* < 0.01) and recovery stage (*p* < 0.001). Finally, fiber supplementation post-antibiotic treatment ([S3]:After) led to improved recovery with an increase in microbial diversity compared to the glucose group (Fig. 1d). Effect size of antibiotics was significantly lower during treatment with fiber supplementation (Fig. 1e) [S1]:Before and [S2]:During. Supplementation of fiber post-treatment reduced antibiotic effects only during recovery (Fig. 1e). Accordingly, we found that supplementation with fiber at various stages led to significant changes in microbial composition during treatment. Taxonomic features also indicated a lower microbiome disruption under fiber supplementation (Fig. 1f). In addition to the above data, we observed that supplementing single-purified fibers at 5% composition (Supplementary Fig. 1b) was also beneficial to microbiome recovery post-antibiotic treatment (Supplementary Fig. 1c–f). These observations imply that fine modification to diet can affect microbiome recovery post-antibiotic treatment. From a translational perspective it is particularly beneficial that supplementation at the time of antibiotic administration is as effective as prior to treatment.

### Fiber reduces AID and glucose exacerbates

**Fig. 2:**
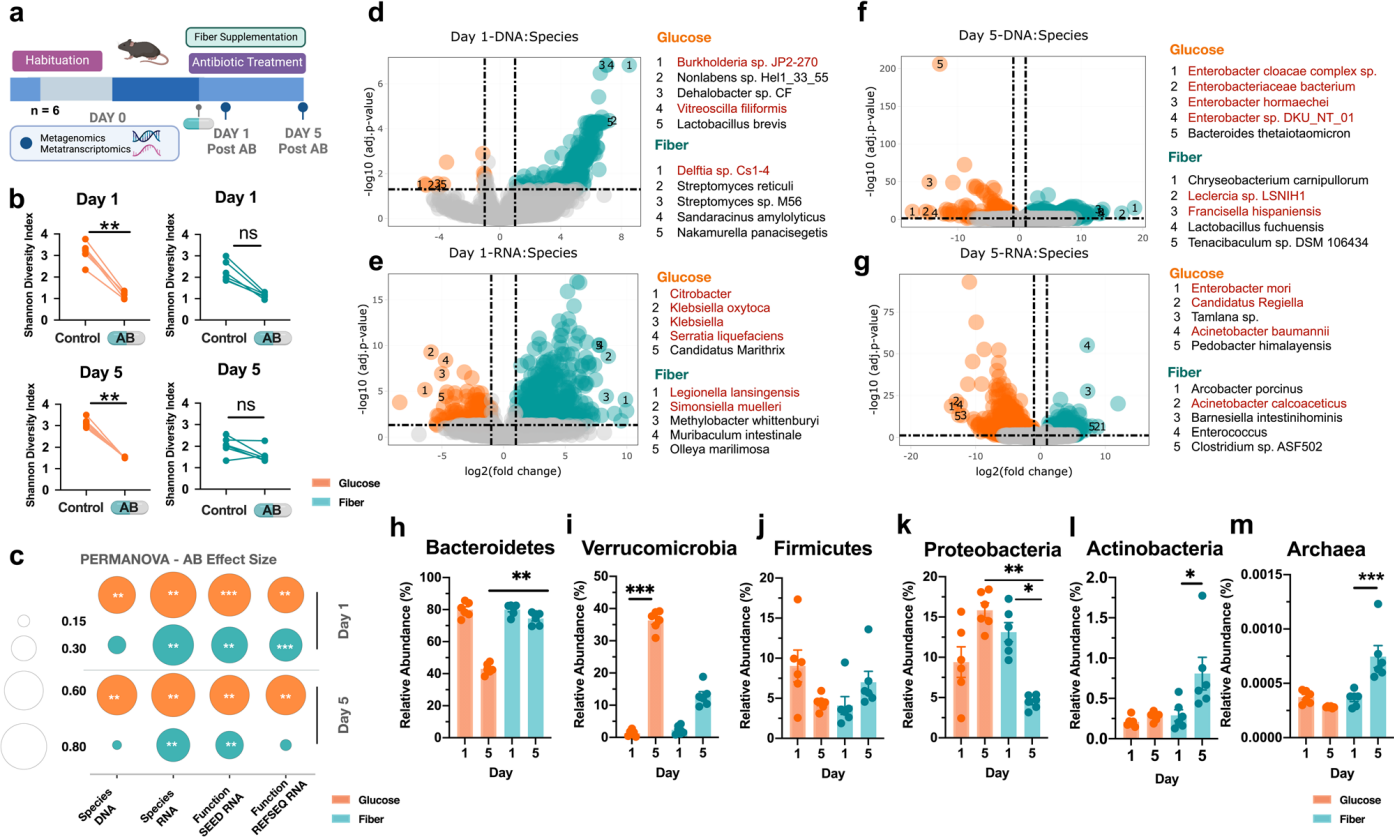
Fiber supplementation reduces antibiotic-induced drop in diversity and Proteobacteria abundance. **a** Mouse experiment schematic (*n* = 6). **b** Antibiotic-induced drop in diversity D1 and D5 during experiment in glucose and fiber-supplemented mice (*n* = 6). Kruskal Wallis with Dunn’s Correction. Day 1 Glucose adj *p* value = 0.0031. Day 5 Glucose adj *p* value = 0.0049. **c** Antibiotic effect size calculated from PERMANOVA analyses of Bray–Curtis distances using the PCoA method from metagenomic and metatranscriptomic data sets (*n* = 6). Plot displays effect size (size of the dot) with significance from adj *p* values denoted by significance stars. Full results shown in source data. DESeq2 of significant species associated with each group day 1 and day 5 of experiment from metagenomic (**d**, **f**) and metatranscriptomic (**e**, **g**) data of antibiotic effect on glucose vs fiber. Proteobacteria species are in red. log2 FC > 1 and p-adj <0.05 (*n* = 6). Full results available in supplementary information and visualized in Rshiny (https://belenkylab.shinyapps.io/shiny). **h** Changes in Bacteroides phylum at D1 and D5 of experiment, adj *p* value = 0.0049. **i** Verrucomicrobia phylum, adj *p* value = 0.0004. **j** Firmicutes phylum **k** Proteobacteria phylum, Glucose day 5 vs Fiber day 5 adj *p* value = 0.0014, Fiber day 1 vs Fiber day 5 adj *p* value = 0.0478. **l** Actinobacteria phylum, adj *p* value = 0.0330. **m** Archaea, adj *p* value = 0.0002. For **h**–**m** (*n* = 6) Mean ± SEM Kruskal Wallis with Dunn’s Correction *p < 0.05; **p < 0.01; ***p < 0.001; ****p < 0.0001.

To expand taxonomic and functional resolution^28,29^ we used metagenomic and metatranscriptomic sequencing of mouse cecal contents day 1 and day 5 post-antibiotic treatment of a replicate experiment in the [S2]:During group (*n* = 6) (Fig. 2a). Mice had non-significant changes in intestinal histopathology day 5 post-antibiotic treatment (Supplementary Fig. 2a, Supplementary Data 1) or bacterial load by day 5 (Supplementary Fig. 2b). Here we also found that mice on the glucose diet had a significantly greater decrease in alpha diversity after antibiotic administration at both timepoints (Fig. 2b). In addition, antibiotic effect size on microbiome composition and function from metagenomic and metatranscriptomic data respectively, had a larger shift on glucose supplemented mice compared to fiber day 1 and day 5 post-antibiotic treatment (Fig. 2c, Supplementary Fig. 2d–g). Glucose supplementation was associated with a greater increase of bacterial species in the Proteobacteria phylum(red) in the metagenomic and metatranscriptomic data, day 1 and day 5 post-antibiotic treatment (Fig. 2d–g) (Supplementary Data 4). By day 5 of the experiment, we observed glucose to have large shifts in species composition and function largely from Proteobacterial species (Fig. 2e, g). In the short-read metagenomic data Proteobacteria and Verrucomicrobia phyla were increased in the glucose supplemented mice (Fig. 2k, I) (Supplementary Data 3) while fiber supplementation led to increases in Archaea and Actinobacteria (Fig. 2m, l). The association with Proteobacteria and dysbiosis^14^ suggests that antibiotic disruption is exacerbated by glucose and limited by fiber. Archaeal species are sensitive to aerobic environments^30^ and their increase in abundance with fiber supplementation suggests fiber helps to maintain gastrointestinal (GI) anaerobicity. We observed similar patterns of taxonomic changes using de novo gene assembly of short-read metagenomic data. We assembled 54 high-quality metagenome-assembled genomes (MAGs) across our samples (Supplementary Fig. 3a, b). Using linear discriminant analysis of the relative abundance of MAGs, we identified significant changes in microbiome composition associated with glucose and fiber supplementation during antibiotic treatment 5 days after antibiotic administration (Supplementary Fig. 3g). These shifts agree with short-read analysis (Supplementary Fig. 3c–f) and suggest that there are robust compositional differences between glucose and fiber supplementation during antibiotic treatment.

### Diet has divergent metabolic responses on the gut microbiome: Glucose increases oxidative metabolism and Fiber represses

**Fig. 3:**
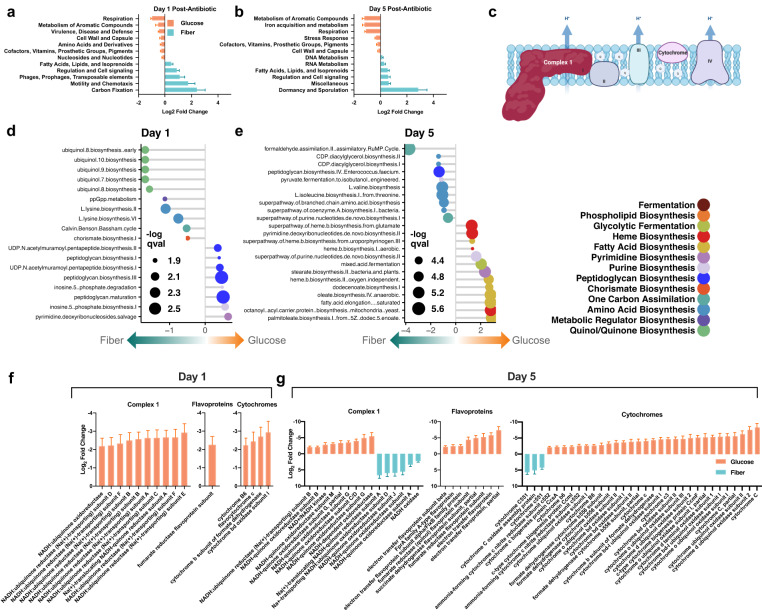
Fiber reduces usage of oxidative metabolism and electron transport chain during antibiotic treatment. DESeq2 analysis of metatranscriptomic dataset aligned to the SEED database (*n* = 6). Significant increases in the glucose (orange) and fiber (blue) diets D1 (**a**) and D5 (**b**) during antibiotic treatment. Log2 FC ± SEM padj < 0.05 and log2 FC > 2. Antibiotic effect on glucose vs fiber is shown. Full results in Supplementary Information. **c** Schematic of proteins involved in bacterial electron transport. Significant pathways increased in fiber (left) and glucose (right) as determined by HUMAnN3.0 and MaAsLin2. Day 1 (*n* = 6) (**d**) and day 5 (*n* = 6) (**e**) qval = FDR, Coefficient shown on x-axis. See Supplementary Information for full results. Significant changes in expression of electron transport proteins (complex 1, flavoproteins, cytochromes) aligned to the Refseq database D1 (*n* = 6) (**f**) and D5 (*n* = 6) (**g**) during antibiotic treatment. padj < 0.0001 and log2 FC > 2. Log2 FC ± SEM. Antibiotic effect on glucose vs fiber is shown. Full results in Supplementary Information.

Prior in vitro and in vivo studies have identified that a change in bacterial metabolism has the capacity to promote tolerance or susceptibility to antibiotics. In general, active metabolism is associated with increased susceptibility while metabolic dormancy confers protection^1,19,21^. To determine changes in metabolic function in the gut microbiome, we used metatranscriptomic sequencing of mouse cecal samples. We observed unique metabolic signatures in the gut microbiomes of mice fed glucose and fiber. Using the SEED subsystem database, we found significant increases in pathways involved in respiratory metabolism in glucose supplemented mice during antibiotic treatment (*p* < 0.05) (Fig. 3a, b). Conversely, fiber supplementation was associated with increased metabolic pathways assigned to dormancy, carbon-fixation and fatty-acid metabolism. This indicates that glucose and fiber have divergent effects on the bioenergetics of gut bacteria, and this may contribute to the observed differences in taxonomic response. Using the HUMAnN3.0 database we identified significant increases in pathways for peptidoglycan biosynthesis at day 1 post-antibiotic treatment (Fig. 3d). These data suggest that gut bacteria supplemented with glucose are entering a peptidoglycan biosynthesis futile cycle with an overactive metabolism. Peptidoglycan cycling has been observed in vitro but here we show that complex microbial communities in vivo follow the same phenomenon^19^. By day 5 of the experiment glucose supplementation led to increased expression of fatty-acid biosynthesis pathways as well as heme biosynthesis (Fig. 3e). Fiber supplementation led to increases in ubiquinol biosynthesis (Fig. 3d) as well as an increase in carbon-fixation pathways annotated as Calvin-Benson-Bassham (Fig. 3d) despite lack of photosynthetic machinery in MAGs (screened protein sequences in Supplementary Data 2). The ribulose monophosphate (RuMP) cycle is another elevated carbon-fixation pathway likely arising from Archaeal methanogenesis which is typically downstream of fiber fermentation^30^ (Fig. 3e). Ubiquinol and heme biosynthesis both assemble iron-sulfur cluster proteins for biochemical reactions, however an increase in heme biosynthesis suggests that the chemical environment contains higher energy electron transfers^31^ potentially from the increased aerobic metabolism. This further adds to the data displaying that glucose supplementation promotes an aerobic inflammatory GI environment.

To further understand this increase in respiratory metabolism, we quantified the gene expression of proteins involved in the electron transport chain (ETC) (Fig. 3c). Using differential abundance analysis of transcriptomic reads aligned to the RefSeq database (Supplementary Data 6), we found that glucose was significantly associated with increased ETC activity during antibiotic challenge.

Expression of complex 1, flavoproteins and cytochromes was greater in glucose supplemented mice post-antibiotic treatment (*p* < 0.0001) (Fig. 3f, g). These iron-sulfur cluster proteins are crucial to the energy-converting electron transfer reactions that bacteria utilize for energy^15^. The overall decrease in these transcripts in the fiber diet suggests that this community has less respiratory metabolism. As an internal control we utilized RNA polymerase subunit beta as a house-keeping gene and found no-significant differences between any groups (Supplementary Data 5).

We quantified antibiotic resistance gene(ARG) expression in our treatment groups and found that the fiber diet had greater ARG expression from Proteobacterial species compared to glucose (Supplementary Fig. 4). However, despite this ARG expression pattern Proteobacteria abundance decreases by day 5 of the experiment strengthening the role of bacterial metabolism in the observed phenotypes.

### Fiber supplementation increases fermentative metabolism and buffers gut redox potential

**Fig. 4:**
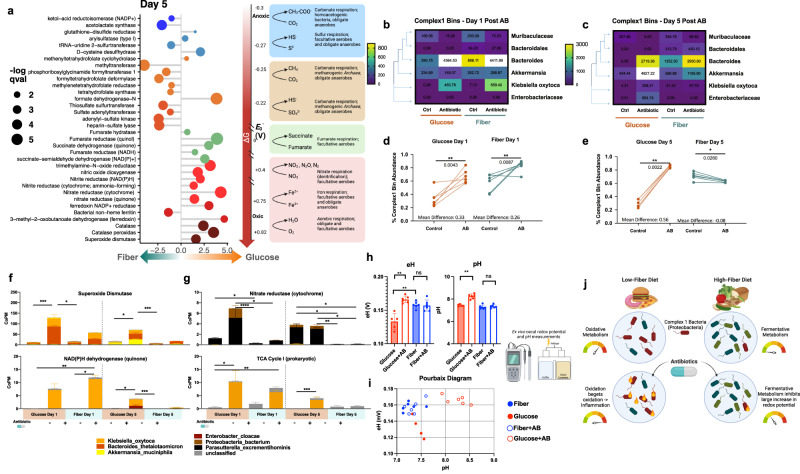
Fiber reduces signatures of high redox metabolic activity and reduces abundance of complex 1 bacteria. (**a**) Significant differences in HUMaN3 reaction expression during D5 of experiment across the redox tower as determined by MaAsLin2. Coefficient shown on x-axis and size of dot represents *q*-val = FDR. Full results in Supplementary Information. (**b**) Heat map depicting abundance of MAGs containing complex 1 D1 and D5 (**c**) during experiment. White boxes represent values outside of the scale. Change in abundance of complex 1 MAGs shown with two-tailed Mann–Whitney for significance D1, glucose *p* value = 0.0043, fiber *p* value = 0.0087 (**d**) and D5, glucose *p* value = 0.0022, fiber *p* value = 0.0260 (**e**) of experiment (*n* = 6) *p < 0.05; **p < 0.01; ***p < 0.001; ****p < 0.0001. (**f**) Changes in expression of superoxide dismutase, adj *p* values left to right = 0.0009, 0.0023, 0.0374, 0.0005. Changes in expression of NAD(P)H dehydrogenase (quinone), adj *p* values left to right = 0.0033, 0.0119, 0.0268, 0.0006. (**g**) Changes in expression of Nitrate reductase (cytochrome), adj *p* values left to right = 0.0478, <0.0001, 0.0478, 0.0240, 0.0449, 0.0081, 0.0161. For **f**, **g**: (*n* = 6) Copm = copies per million reads. Mean ± SEM Kruskal Wallis with Dunn’s Correction *p < 0.05; **p < 0.01; ***p < 0.001; ****p < 0.0001. (**h**) eH and pH values from additional mouse experiment (*n* = 6). Mean ± SEM Significance determined by two-tailed Mann–Whitney test, *p* values left to right = 0.0080, 0.0078, 0.0080. (**i**) Pourbaix diagram depicting eH and pH values from lyophilized cecal contents of mice with and without antibiotics measured within 24 h after rehydration with RO water. (**j**) Conclusion schematic.

To better elucidate the mechanism behind this metabolic shift in response to the tested diets, we used HUMAnN3.0 and MaAsLin2 to identify changes in biochemical processes across the bioenergetic scale associated with diets under antibiotic treatment. Disentangling metabolism and bioenergetics in the gut microbiome is challenging due to the limited understanding of bacterial biochemistry of the many unculturable species in the gut. However, large shifts in bacteria with varying functions in the metabolic ecology of the gut may accurately predict gut biochemistry^8,11,32^. In this study, we searched our metatranscriptomic dataset for biochemical reactions based on electron acceptors and redox potential. We observed significantly increased transcription of pathways involving oxygen and nitrate as terminal electron acceptors post-antibiotic treatment (Fig. 4a) in the glucose diet compared to the fiber diet. We also observed increased respiration and ETC activity indicating more oxidative metabolism (Fig. 4f, g) (Supplementary Fig. 6a, Supplementary Data 8). This increase in gut redox potential thermodynamically selects for increased respiratory activity and restricts biochemical activity of bacteria that predominantly rely on fermentative metabolism. Multiple studies have found that gut commensals associated with improved health utilize fermentation to create short-chain fatty acids and maintain an anaerobic environment in the gut^33,34^. We found that fiber supplementation was associated with increased expression of carbohydrate active enzymes^34^ (CAZymes) that were involved in polysaccharide degradation. The fiber cocktail was associated with increased expression of total CAZymes as well as fiber-specific CAZymes involved in the degradation of pectin and inulin (Supplementary Fig. 5a, b). This indicates that the microbiome of fiber-supplemented mice has increased expression of enzymes that could contribute substrates for fermentation.

Linear discriminant analysis identified similar metabolic signatures at the pathway level. Fiber is associated with increased carbon-fixation pathways classified as Calvin-Benson-Bassham cycle (Supplementary Fig. 7b, Supplementary Data 7) and pathways involved in increased production of Coenzyme A (Supplementary Fig. 7b, d) which could be another indicator of increased carbon fixation. In addition, metabolic pathways unique to an anaerobic environment were significantly associated with the fiber group suggesting that this diet reduces oxygen in the gut and protects from respiratory metabolism. This increase in carbon fixation could be due to increased CO_2_ over O_2_ as suggested by a recent study exploring the reverse TCA cycle pathways found in bacteria^35,36^. These data contrast with the aerobic oxidative pathways induced in the glucose diet. Catabolic oxidative pathways such as glycolysis, TCA, and the pentose phosphate pathway (Supplementary Fig. 7a, c) were associated with the glucose diet by day 5 of the experiment. In short, the glucose diet is more associated with catabolic oxidative metabolism while the fiber diet was associated with anabolic reductive metabolism. This suggests that fiber supplementation can encourage protective fermentative metabolism by reducing gut redox potential and oxygen and leading to protection from the damaging respiratory metabolism seen post-antibiotic treatment.

### Glucose increases abundance of complex 1 bacteria post-antibiotic treatment

The transcriptomic data suggest that ETC activity, specifically complex 1, is involved in the metabolic shift observed in our data. Recent phylogenomic studies have found that ~50% of bacteria have complex 1^37,38^, and these are mostly in the Proteobacteria phylum. Presence of complex 1 can be an indication of their bioenergetic capacity. Complex 1 contains large iron-sulfur clusters making this protein responsible for high-energy electron transfers. Based on the observed significant increase in genes for subunits of complex 1 (Fig. 3f, g) (Supplementary Fig. 6a), and the larger shift in redox potential (Fig. 4a), we hypothesized that glucose may be driving the community composition for increased abundance of complex 1 bacteria. To understand how the metabolic shift observed in the respective diets was contributing to the community composition, we used Phylophlan3.0 to search our 54 MAGs for presence of complex 1. We found 6 MAGs in our dataset that we determined to contain complex 1 and observed significant changes in their relative abundance. Compared to the abundance of complex 1 in all bacterial genomes, the anaerobic environment of the gut likely explains the lower abundance in our samples (6/54). We found that both diets led to a spike in complex 1 bacteria 1-day post-antibiotic treatment (Fig. 4b, d) specifically subunit I (Supplementary Fig. 6). However, by day 5 of the experiment the glucose diet continued to have greater abundance of complex 1 bacteria compared to the control while the fiber diet had a decrease (Fig. 4c, e) (Supplementary Fig. 6). It is important to note that this increase was limited to 4 of the 6 MAGs identified as containing complex 1. MAGs classified to Muribaculaceae and Bacteroidales did not exhibit an antibiotic-induced increase in abundance. Although presence of complex 1 can improve survival in a high redox environment, many other factors can play a role in the fitness of a bacterium such as antibiotic resistance genes, growth rate and competition for carbon sources. The environment of the gut also creates chemical gradients and has specific spatial organization of bacteria that drive composition^11,12^. Due to this heterogeneity, the redox environment may not have equal effects on all bacteria found in the gut. Here, we identify that glucose supplementation can increase gut redox driving the microbiome composition to contain more complex 1 bacteria compared to a fiber-supplemented diet. These bacteria are major contributors to the metabolic shift towards aerobic respiratory metabolism seen with glucose supplementation during antibiotic treatment (Fig. 4f, g) (Supplementary Fig. 8a–d).

### Fiber supplementation protects from antibiotic-induced increase in gut redox

Thus far we have relied on sequencing methods to understand the gut redox environment on glucose and fiber diets. To assess if the observed changes in our metagenomic and metatranscriptomic data translate to physiological changes in the gut we used published methods to measure the chemical redox potential in the cecal contents of our mice^4,32,39–41^. We first validated these methods on mice given a standard chow diet and found that antibiotics increased the chemical redox potential (Supplementary Fig. 9a–f). These results corroborate with other studies measuring the effect of antibiotics on redox potential^4^. We then measured the chemical redox potential of cecal contents from mice given our glucose and fiber diets 5 days post-antibiotic treatment. We chose this time point based on the sequencing data which suggested large changes in complex 1 utilization by day 5 of the experiment. We found that only the glucose diet post-antibiotic treatment was associated with a significant increase in gut redox potential (Fig. 4h) (Supplementary Fig. 9g–i). Because redox potential (eH) is also affected by the pH of the environment^32^, we measured these in parallel and mapped the data on a Pourbaix diagram (Fig. 4i). These data suggest that diet drastically alters the chemical environment of the gut contributing to changes in the biochemical activity of gut microbes. ATP measurements from these samples showed non-significant changes between control and antibiotic treated groups (Supplementary Fig. 9j, k). Antibiotic-induced changes in the chemical environment of the gut were more significant on the glucose diet compared to the fiber diet indicating the protective capacity of fiber to buffer gut redox.

The metabolism-driven mechanism of susceptibility proposes that active catabolic metabolism in bacteria can affect their susceptibility to antibiotics^21^. Most microbial studies thus far have documented this phenomenon in vitro. In this study, we explore the link between metabolism and antibiotic susceptibility in the complex population of the gut microbiome. We show how dietary inputs to the gut microbiome can alter the biochemical output of microbes coinciding with broad changes to the gut chemical environment. Specifically, we observe that altering bacterial metabolism through dietary supplementation with fiber can protect from negative antibiotic effects (Fig. 4j).

Our work demonstrates large diet-dependent metagenomic, metatranscriptomic and chemical shifts in microbiome structure and function during antibiotic treatment. The multi-omic methods employed in this study are well-established in the field, however chemical measurements of the microbiome are still in their early stages. While there are limited studies measuring gut redox potential and pH, these methods require further improvement. Ideally, redox potential should be measured over time within the gut environment. This is only possible with in vivo wireless sensors as shown in Baltsavias et al.^32^. Further studies to elucidate the dynamics of redox potential changes in response to diet modification are required to understand the detailed mechanisms involved in antibiotic protection. Intrinsic to any dietary modulation, removing a component like fiber requires the addition of a replacement in order to maintain equivalent nutritional composition. It is difficult to identify an ideal replacement as most animal-safe diet additives can be metabolized by the host or the microbiome. In this publication, we chose glucose as the low-fiber supplement. However, it is important to note that this is not a true control but rather a contrasting fiber-free dietary condition^25,26^.

Additionally, we focus this study on the activity of gut microbes and do not elucidate the role of host contribution to GI chemical environment. A diet low in fiber and high in sugars has been previously described to be harmful to the host, leading to increased oxygen in the GI as well as changes in immune response. This has been shown to decrease vaccine efficacy^42^ in humans suggesting that diet has important immunological impacts. These studies suggest that glucose alone has vast physiological effects on the host that can affect GI environment. In this study, we do not identify if changes in the gut environment are elicited directly by the diet component, host processing of the diet, or through activity of the microbiota on the diet.

However, we do not see significant differences in tissue morphology or cytokine production (Supplementary Fig. 10) between groups potentially due to the length of the experiment being limited to 5 days. Although there are non-significant differences in morphology or cytokine production, there are likely metabolic consequences to host cells as a result of the glucose supplementation that can indirectly affect microbiome function. We have included additional differential expression analyses of our ‘omic data to compare microbiome effects elicited by the diets alone without antibiotics, and have added this data to an interactive Rshiny app (https://belenkylab.shinyapps.io/shiny). This will allow for better interpretation of the baseline microbiome changes from the respective diets.

This work makes important strides in linking changes in diet-induced redox potential and resulting microbial activity to differential antibiotic susceptibility. The next important steps are to establish causation between changes in redox potential and antibiotic susceptibility in the context of the host. Future studies can target investigation towards the effects of diet directly on host cell metabolism as it relates to microbiome changes and determine if the observed differences translate to male mice. Metabolism is intrinsically a balancing act between growth and the toxic consequences of this activity. We hope that future anti-AID therapies can target this metabolic balance to achieve optimal therapeutic outcomes without microbiome related morbidity.

## Methods

### Experimental model and subject details

#### Mice

Experimental procedures involving mice were all approved by the Institutional Animal Care and Use Committee of Brown University under IACUC Protocol Number 1706000283. Four-week-old female C57BL/6J mice were purchased from the Jackson Laboratories (Bar Harbor, ME, USA). Mice were habituated for two-weeks following their arrival at Brown University. All animals were cohoused together in specific-pathogen-free (SPF), temperature controlled (21 + 1.1 °C), 30–70%v humidity, and 12 h light/dark cycling conditions. Mice were randomized into new cages following the habituation period. Mice were given the specified diets in powdered form. Mice used in (Supplementary Fig. 9a–f) for redox potential measurements were given the typical laboratory chow (Laboratory Rodent Diet 5001, LabDiet, St. Louis, MO, USA).

#### Diet design

Purified diets were designed with veterinarians from Envigo-Teklad (Madison, WI, USA) based on fiber content present in the typical mouse laboratory chow. The diet is based on the widely used purified AIN-93G(TD.180901) diet and modified to contain reduced carbohydrates with the cellulose completely removed and the cornstarch reduced. Additionally, the cornstarch supplied in the diet (Buffalo cornstarch, Envigo-Teklad) is modified to be more host accessible reducing its prevalence in the cecum and lower GI tract. This diet was custom designed at 80% composition to allow for 20% (w/w) supplementation with other carbon sources without affecting protein, fat, vitamin and mineral ratios. The diet is powdered and irradiated and given to mice in feeding jars. Mice were allotted 5 g/mouse per day in the jars and the food was replenished daily in new autoclaved feeding jars. This quantity was determined after conversations with Envigo-Teklad veterinarians and is well over the typical amount consumed by mice. The glucose diet was used a no fiber condition in all experiments and supplemented with 20% glucose (Fisher Scientific). The fiber diet contains 20%(w/w) supplementation of a custom fiber cocktail including inulin (15%) (Chem-Impex), pectin (15%) (MP Biomedicals), dextrin (15%) (Sigma-Aldrich), levan (15%) (Realbiotech CO., Ltd), arabinoxylan (20%) (Anthony’s Organics), beta-glucan (25%) (Anthony’s Organics), cellulose (10%) (EMD Millipore). For single-purified fiber supplementation in Supplementary Fig. 1 a 95% composition of the same diet was used with 5% (w/w) supplementation of pectin or inulin.

#### Animal experiments

C57BL/6J mice following the habituation period were given 1 week to acclimate to the 0-fiber diet with 20% (w/w) glucose. Following this diet acclimation period mice were randomized into new cages for each diet/antibiotic condition. Amoxicillin was administered via drinking water (25 mg/kg/day) ad libitum for the specified timepoints. All drinking water was filter-sterilized prior to administration. Fecal samples were collected at the specified timepoints and stored at −20 °C until nucleic acid extraction. Cecum contents were collected directly into bead-bashing tubes with DNA/RNA shield (Zymo Research (Irvine, CA, USA) and stored at −80 °C for nucleic acid extraction. For the eH, pH measurements total cecal contents were immediately flash frozen in liquid nitrogen and lyophilized and stored at −80 °C until measurements were taken.

#### Nucleic acid extraction and quantification

Total nucleic acids (DNA and RNA) were extracted from samples using the ZymoBIOMICS DNA Miniprep Kits from Zymo Research (Irvine, CA, USA) using the extraction protocols as per the manufacturer instructions. For fecal samples the Fecal 96 Zymo DNA Extraction kit was used. For DNA/RNA parallel extraction the Zymo Magbead DNA/RNA kit was used. Total DNA was eluted in nuclease-free water and quantified using the dsDNA-HS on a QubitTM 3.0 fluorometer (Thermo Fisher Scientific, Waltham, MA, USA) before use in amplicon/library preparations.

#### 16S rRNA amplicon preparation and sequencing

The 16S rRNA V4 hypervariable region was amplified from total DNA using the barcoded 515F forward primer and the 806R reverse primers from the Earth Microbiome Project^43^. Amplicons were generated using 5X Phusion High-Fidelity DNA Polymerase under the following cycling conditions: initial denaturation at 98 °C for 30 s, followed by 25 cycles of 98 °C for 10 s, 57 °C for 30 s, and 72 °C for 30 s, then a final extension at 72 °C for 5 min. Gel electrophoresis was used to visualize amplicons and pooled in equimolar amounts. The pooled amplicon library was submitted to the Rhode Island Genomics and Sequencing Center at the University of Rhode Island (Kingston, RI, USA) for sequencing on the Illumina MiSeq platform. Amplicons were paired-end sequenced (2 × 250 bp) using the 600-cycle kit with standard protocols.

### 16S sequencing analysis

Raw 16S rRNA reads were first demultiplexed with idemp. Quality filtering, trimming, denoising with DADA2^44^ (q2-dada2), and merging using the Qiime2 pipeline (version 2019.10)^45^. Ribosomal sequence variants were aligned with mafft^46^ (q2-alignment), and phylogenetic tree construction was done with fasttree2^47^ (q2-phylogeny). Taxonomic assignment was conducted using the pre-trained Naive Bayes classifier and the q2feature-classifier^48^ trained on the SILVA 132 99% database^49^. Alpha diversity (Shannon, Faith’s phylogenetic diversity) and beta diversity (Bray-Curtis dissimilarity) were calculated using the phyloseq package^50^ (version 1.30.0) in R (version 3.6.2)^50,51^.

#### Metagenomic and metatranscriptomic library preparation

Metagenomic and metatranscriptomic sequencing libraries were prepared as described in ref. ^2^ Cabral 2020. For metagenomic libraries 100 ng of DNA was used with the NEBNext® Ultra II FS DNA Library Prep Kit (New England BioLabs, Ipswich, MA, USA) as per manufacturer’s instructions to generate a pool of fragments at 300 bp ± 50 bp. Metatranscriptomic libraries were created with total RNA (1 μg) using the MICROBExpress kit (Invitrogen, Carlsbad, CA, USA), NEBNext® rRNA Depletion Kit for Human/Mouse/Rat (New England BioLabs, Ipswich, MA, USA), and the NEBNext® Ultra II Direction RNA Sequencing Prep Kit as per the manufacturers’ instructions to generate a pool of fragments at 300 bp ± 50 bp. Metagenomic and metatranscriptomic libraries were pair-end sequenced (2 × 150 bp) on the Illumina HiSeq X Ten. An average of 12,928,385 reads per metagenomic sample and 52,848,755 reads per metatranscriptomic sample. A control metagenomic sequencing library was made using the Zymobiomics Microbial Community Standard (D6300, Zymo Research) (Irvine, CA United States) and added to the sequencing run. This data was used to validate the accuracy of the sequencing run. Sequencing of this standard resulted in relative abundances near the theoretical composition with all community members identified.

#### Metagenome assembly

Metagenome-assembled genomes from the metagenomic reads were constructed using the metaWRAP pipeline^52^. Raw reads were processed using the READ_QC module with FASTQC 0.11.8 and TrmGalore 0.5.0. Assembly was done with the ASSEMBLE module in metaWRAP with metaSPAdes 3.13.0^53^ and MegaHit 1.1.3^54^. Assembled contigs were binned with the BINNING module using CONCOCT 1.0.0, MaxBin2 2.2.6^55^ and metaBAT2 2.12.1^56^. The BIN_REFINEMENT and BIN_REASSEMBLY module was used to consolidate bins and select bins with greater than 90% completion and less than 10% contamination to attain 54 high-quality MAGs. Quantification was done using the QUANT_BINS module with SALMON 0.13.1^57^ and classified with the CLASSIFY_BINS module and taxator-tk 1.3.3e. To improve bin classification the CAT and BAT 2021-01-07 tool^58–60^ was also used.

#### MAGs phylogenetic tree construction and complex 1 identification

PhyloPhlAn 3.031 was used to construct a phylogenetic tree from the assembled MAGs using the high diversity option and the PhyloPhlAn database. To identify MAGs with complex 1 a custom phylophlan database was constructed using the Uniref90 sequence clusters for each of the 14 subunits (A–N) of the bacterial NADH-quinone oxidoreductase. Supplementary Data 2 contains the Cluster ID and size information. PhyloPhlAn was then run using this database to construct a phylogenetic tree of MAGs to identify complex 1 MAGs.

#### Metagenomic and metatranscriptomic short-read processing

Raw metagenomic and metatranscriptomic reads underwent trimming and decontamination using KneadData (version 0.6.1) as previously described 1,2,32. Illumina adapter sequences were removed using Trimmomatic 56 (version 0.36), then depleted of reads that mapped to C57BL/6J, murine mammary tumor virus (MMTV, accession NC_001503) and murine osteosarcoma virus (MOV, accession NC_001506.1) using Bowtie2 (version 2.2) 1,57. Metatranscriptomic reads were additionally depleted of sequences that aligned to the SILVA 128 LSU and SSU Parc ribosomal RNA databases as previously described 1,2.

#### Short read classification

Classification of metagenomic reads was done with NCBI RefSeq using Kraken2 (version 2.0.7-beta, “Kraken2 Standard Database”) with a k-mer length of 35 (Wood et al.^61^). Bracken (version 2.0.0) was then used to calculate phylum- and species-level abundances from Kraken2 reports, and the R package phyloseq (version 1.28.0) was used to calculate a- and b-diversity metrics^62^.

#### Metatranscriptomic analysis: SAMSA2

A modified version of the Simple Annotation of Metatranscriptomes by Sequences Analysis 2 (SAMSA2) pipeline to annotate trimmed and decontaminated reads as previously described^1,63,64^. This modified pipeline uses Paired-End Read Merger (PEAR) utility to merge reads and DIAMOND (version 0.9.12) aligner algorithm^62,65^ to align to the RefSeq, SEED Subsystem, and CAZyme databases^34,66^.

#### Metatranscriptomic analysis: HUMAnN3

HUMAnN3^28^ was used to identify changes in gene expression from cleaned metatranscriptomic and metagenomic reads. Reads were aligned to the UniProt/UniRef 2019_01 databases to identify expression of reactions and MetaCyc to identify expression of pathways. MetaPhlAn 3.0^28^ and the ChocoPhlan pangenome database was used to classify reads to bacterial species. Reads aligning to reactions and pathways are normalized to sequencing coverage and reported as copies per million (cpm, Copm) in the metatranscriptomic and metagenomic samples.

#### Redox potential and pH measurements

Redox potential was measured according to a modified protocol^67^ from^32,40,41^ with an Ag/AgCl Reference electrode (Radiometer analytical E21M002, Radiometer Analytical Pt plate electrode 5 × 5 mm M241 Pt), and a voltmeter. Flash frozen and lyophilized cecal contents were first rehydrated at 1:10 ratio in RO water and vortexed for 10 min with 10-min breaks over 60 min. The samples were blinded and measured in random order. These extracts were then used to measure eH and pH within 24 h (Fig. 4) or 96 h (Supplementary Fig. 9g–i). Each sample was vortexed for another minute prior to measurement. A 0.1 M KCL agar plate was used as the base for the samples. A cut plastic pipette tip was inserted into the agar to hold 300 μL of the cecal extract. The platinum electrode was inserted into the sample and the reference electrode was inserted into the 0.1 M KCL agar. The redox potential was allowed to stabilize for 20 min (Fig. 4) or 15 min (Supplementary Fig. 9g–i) before recording the voltage. Electrodes were cleaned between each measurement and placed in the RENOVO cleaning solution (Hach, Loveland, CO, USA) for 1 min. The electrode was then rinsed with RO water and used to measure a 220 mV redox buffer solution to validate integrity of the electrode before measuring the next sample. The same cecal extracts were used to measure pH using a pH meter and for subsequent validation with ATP assays, 16S rRNA sequencing and qPCR bacterial load.

#### ATP assay

Cecal extracts from the eH and pH measurements were used to measure ATP with the BacTiterGlo Microbial Cell Viability Assay (Promega, Madison, WI, USA) as per manufacturer’s instructions. A standard curve was also measured in each assay to validate assay methods.

#### qPCR for bacterial load

Quantitative PCR for bacterial load determination was done as described previously in Vasihnava et al. Q-PCR analysis of bacterial genomic DNA using iTaq Master Mix (BioRad, Hercules, CA, USA) and universal 16S rRNA gene primers. A standard was constructed with reference to cloned bacterial DNA corresponding to a 179 bp section of the 16S rRNA gene that was amplified using 16s RNA specific primers. Sq values were normalized to the amount of DNA in the sample.

#### Serum cytokine panel

After animal sacrifice, whole blood was obtained by cardiac puncture and placed in a microcentrifuge tube to coagulate for 30 min. The collection tubes were then centrifuged at 13,000 × *g* for 10 min to separate the serum, which was then transferred to a new microcentrifuge tube and frozen at −80 °C until further processing. When ready, the samples were thawed on ice and divided into a working aliquot and a re-frozen stock aliquot. The working aliquot was analyzed for signs of inflammation in mice using the LEGENDplex Mouse Inflammation Panel (13-plex) (BioLegend, San Diego, CA) flow cytometry kit, following the manufacturer’s instructions. The samples were analyzed on the Attune NxT Flow Cytometer (ThermoFisher, Waltham, MA) and then evaluated using the LegendPlex cloud software tool (BioLegend, San Diego, CA).

### Statistical analysis

Specific details of the statistical analyses for all experiments are outlined in the figure legends and Results section. All sample numbers represent biological replicates. PERMANOVA was calculated using the adonis method on Bray-Curtis distance matrices calculated from multidimensional scaling of sequencing data using phyloseq. Control samples were compared against antibiotic treated in each group to determine antibiotic effect size. LEfSe (version 1.0) was used to analyze HUMAnN3 outputs on the Galaxy web server using default settings (http://huttenhower.sph.harvard.edu/galaxy). Metatranscriptomic outputs generated by SAMSA2 were subjected to differential abundance testing using the DESeq2 package (1.24.0) in R (version 3.5.2) under default parameters and included contrast:interaction comparisons^68^. All DESeq2 results were corrected using the Benjamini–Hochberg method (FDR = p-adj) to account for multiple hypothesis testing. ANOVA, unpaired t tests, and Mann–Whitney *U*, Kruskal Wallis tests were performed in Prism GraphPad (version 9.0) without sample size estimation. MaAsLin2 was used to identify significant pathway and reaction annotations from HUMAnN3 outputs. FDR = −log(qval).

### Supplementary information

Supplementaty Information

Peer Review File

Description of Additional Supplementary Files

Supplementary Data 1

Supplementary Data 2

Supplementary Data 3

Supplementary Data 4

Supplementary Data 5

Supplementary Data 6

Supplementary Data 7

Supplementary Data 8

### Source data

Source Data

## Data Availability

The short-read metagenomic and metatranscriptomic sequencing data generated in this study have been deposited in the NCBI SRA database. The metagenome-assembled genomes (MAGs) have been deposited to NCBI GenBank. The 16s sequencing data has been depositing to NCBI SRA. BioProject accession code for all sequences associated with this study is PRJNA984334. The DESeq2, LDA, and MaAsLin2 results generated in this study are provided in the Supplementary Information. Source Data contains all PERMANOVA stats information. Databases used in this study (MMTV, accession NC_001503), (MOV, accession NC_001506.1), SILVA 128 LSU and SSU Parc ribosomal RNA databases (https://www.arb-silva.de/documentation/release-128), RefSeq (10.1093/nar/gkt1274.), SEED Subsystem (10.1093/nar/gkt1226), CAZyme databases (10.1093/nar/gkn663), UniProt/UniRef 2019_01 databases (10.1093/bioinformatics/btm098), SILVA 132 99% database (https://www.arb-silva.de/download/archive/qiime). Source data are provided with this paper.
