# Supplementary material for: Fiber supplementation protects from antibiotic-induced gut microbiome dysbiosis by modulating gut redox potential
Source: Nat Commun. 2023 Aug 24;14:5161. doi: 10.1038/s41467-023-40553-x (PMC10449846; doi:10.1038/s41467-023-40553-x)
Supplement: Supplementary file 1 — Supplementaty Information [file 41467_2023_40553_MOESM1_ESM.pdf]

Fiber supplementation protects from antibiotic-induced gut microbiome dysbiosis by modulating gut redox potential

Penumutchu *et al.*

Supplementary Information

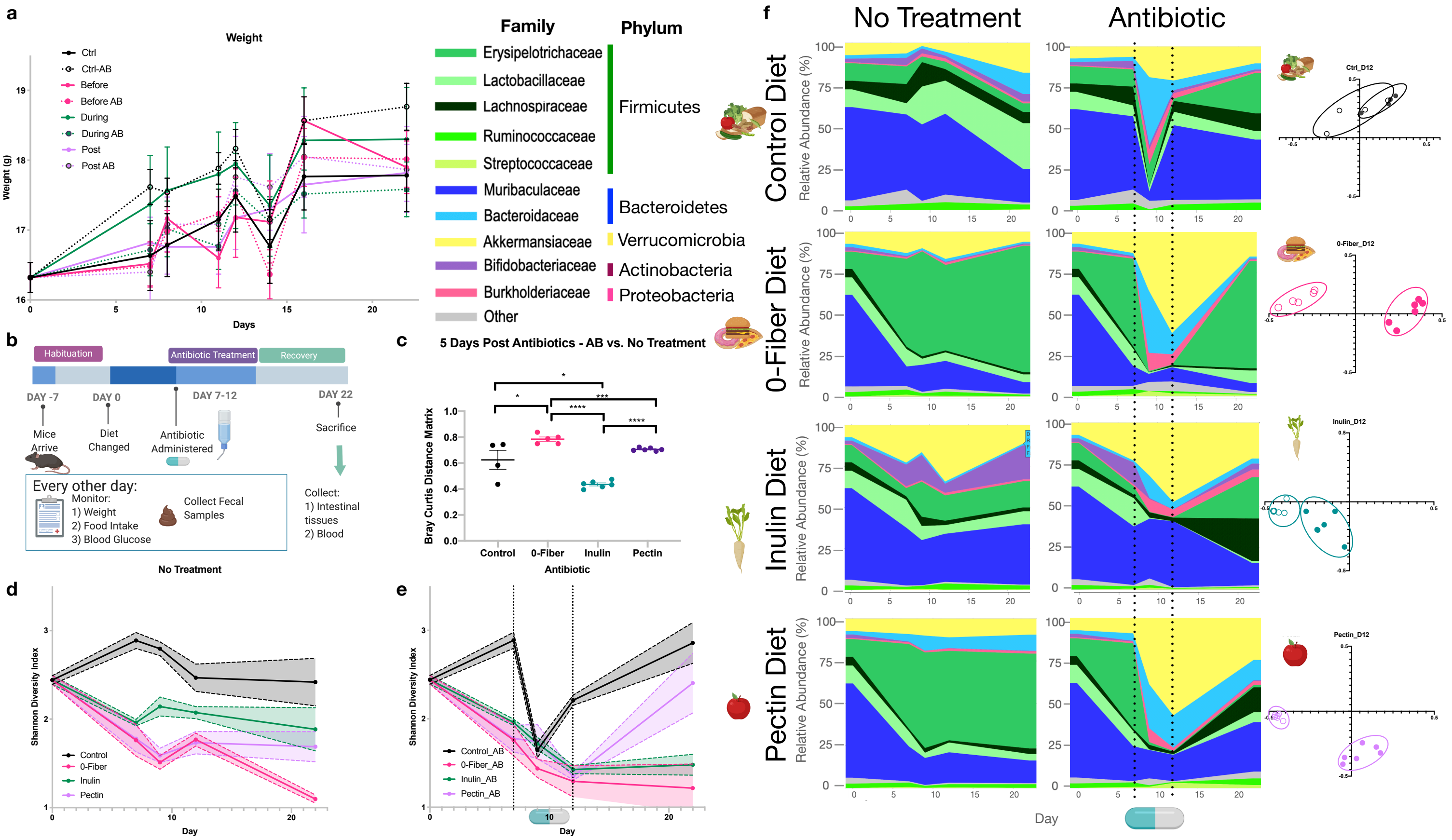

Supplementary Figure 1: (a) Weight of mice throughout the experiment testing stage of fiber supplementation. Mean±SEM (n=6) (b) Mouse experimental timeline for 5% Supplementation of Inulin and Pectin (c) Bray-Curtis values of beta diversity 5 days post antibiotics. (n=6) Mean±SEM. Two-tailed unpaired t-test to compare groups. P values left to right: 0.0476, 0.0128, 0.0008, <0.0001, <0.0001. Shannon diversity values throughout the experiment in control (n=6) Mean±SEM (d) and antibiotic treated mice (e). (f) Relative abundance of bacterial families in fecal samples collected from mice in each diet group. Bray-Curtis PCoA D5 after Antibiotics. Significance determined by unpaired t-test. ∗p < 0.05; ∗∗p < 0.01; ∗∗∗p < 0.001; ∗∗∗∗p < 0.0001. Figure made with BioRender.

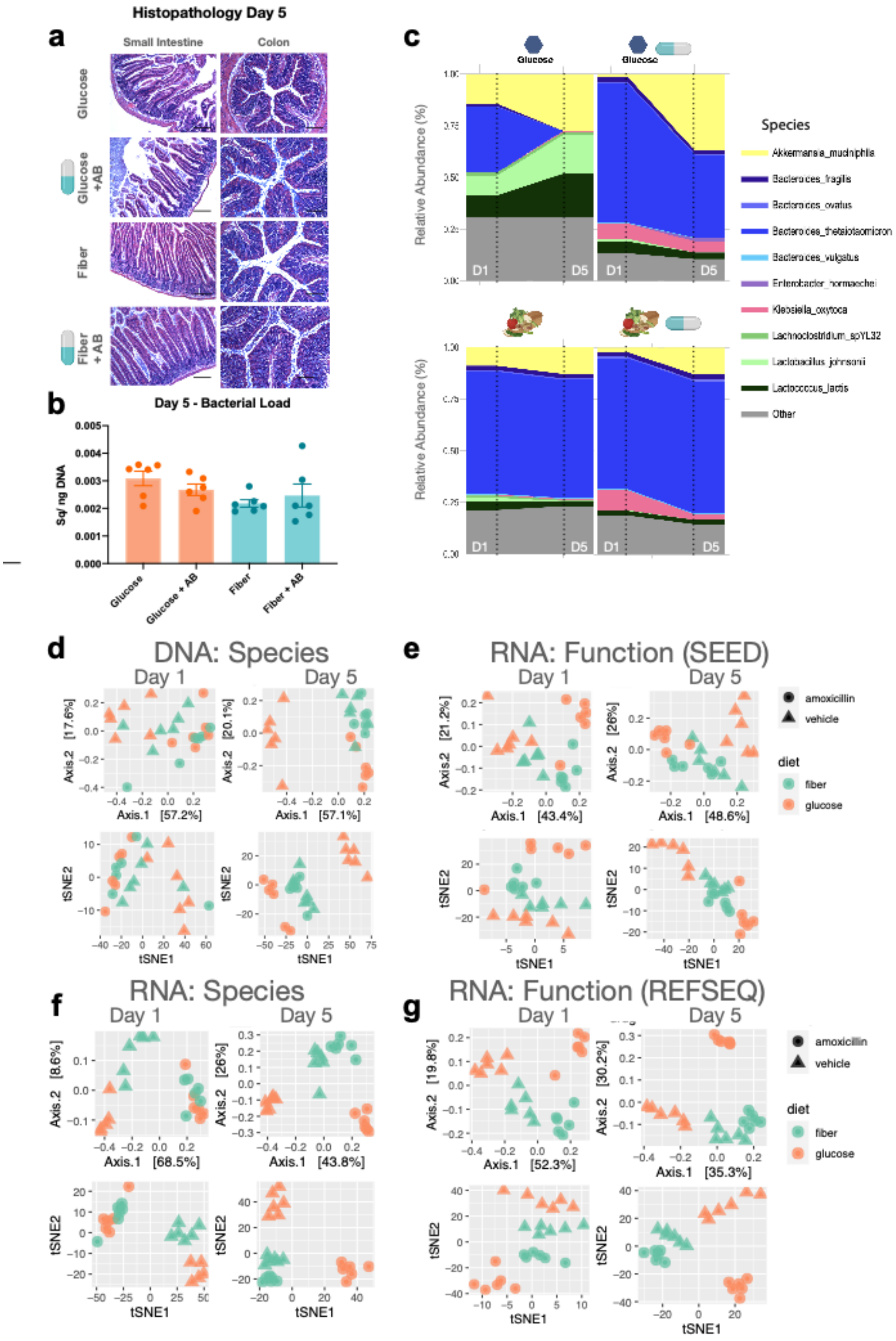

Supplementary Figure 2: (a) H&E-stained mouse colons and small intestines at 20x magnification. Scale bar = 100μm. 6 tissue samples were analyzed per group. Raw pathology scores are displayed in Supplementary Table 1. No significant differences in histopathology were found in blinded analysis by a pathologist. (b) Bacterial load measured by qPCR (n=6) Mean±SEM (D5) No significance determined by unpaired t-test. (c) Relative abundance of bacterial species in short read metagenomic data. PCoA and tSNE plots of Bray-Curtis distance values from DNA:Species (d), RNA:Species (f), RNA:SEED Function data (e), RNA:REFSEQ Function data (g)

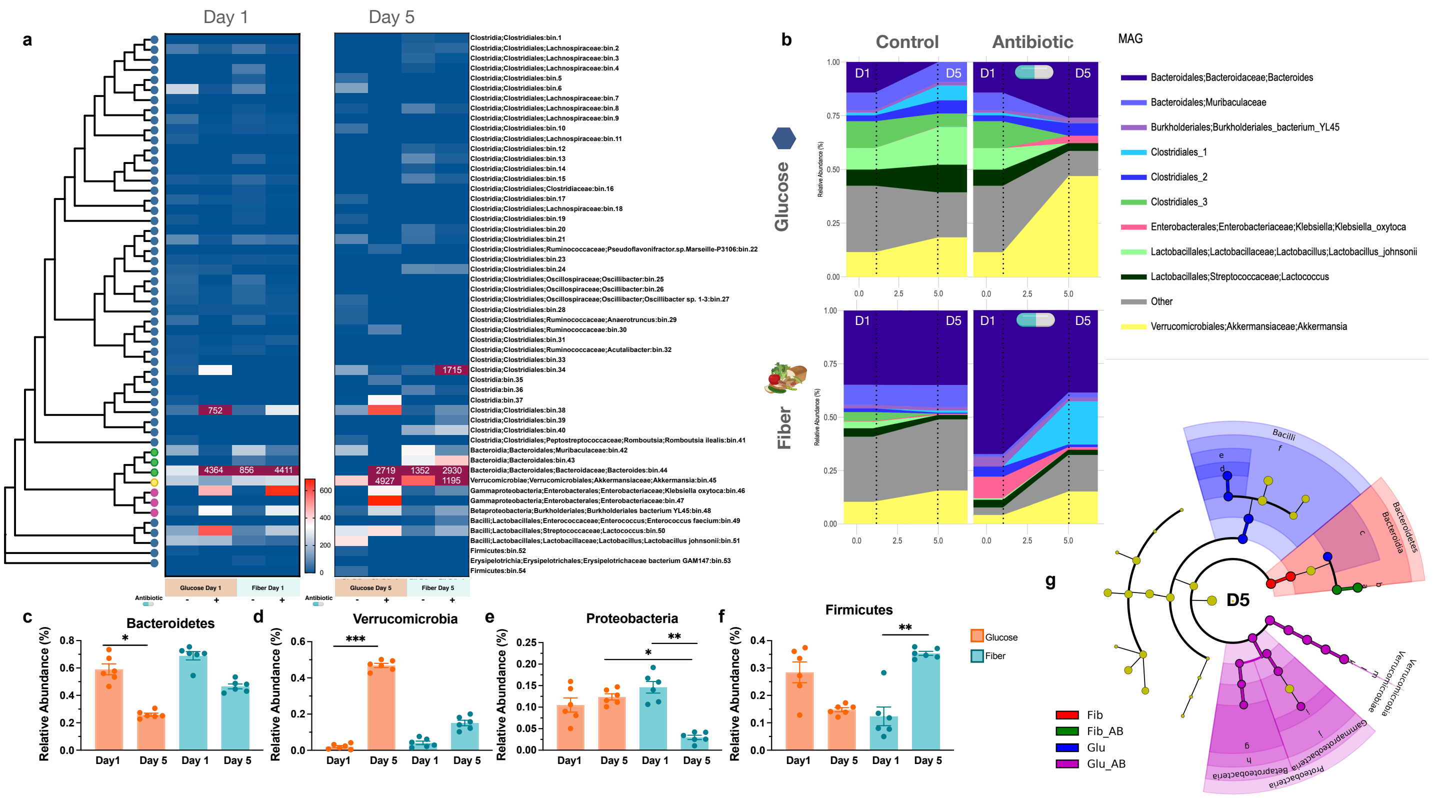

Supplementary Figure 3: (a) Heat map abundance of all 54 MAGS identified with metaWRAP. Phylogenetic tree on left constructed with PhyloPhlAn 3.0. Blue circles for Firmicutes, Green for Bacteroidetes, Yellow for Verrucomicrobia, Pink for Proteobacteria. (b) Relative Abundance of MAGs. (c) Bacteroides MAGs relative abundance. Adj p value = 0.0115. (d) Verrucomicrobia MAGs relative abundance. Adj p value = 0.0003. (e) Proteobacteria MAGs relative abundance. Adj p value = 0.0003. Adj p vales left to right = 0.0256, 0.0012. (f) Firmicutes MAGs relative abundance. Adj p value = 0.0023. (c-f) (n=6) Mean±SEM. Kruskal Wallis with Dunn's Correction $*p < 0.05$; $**p < 0.01$; $***p < 0.001$; $****p < 0.0001$.(g) LDA of significant phyla associated with each group Day 5 of experiment as determined LEfSe. Shown as Cladogram. Cutoffs: LDA > 3 and p-value < 0.05.

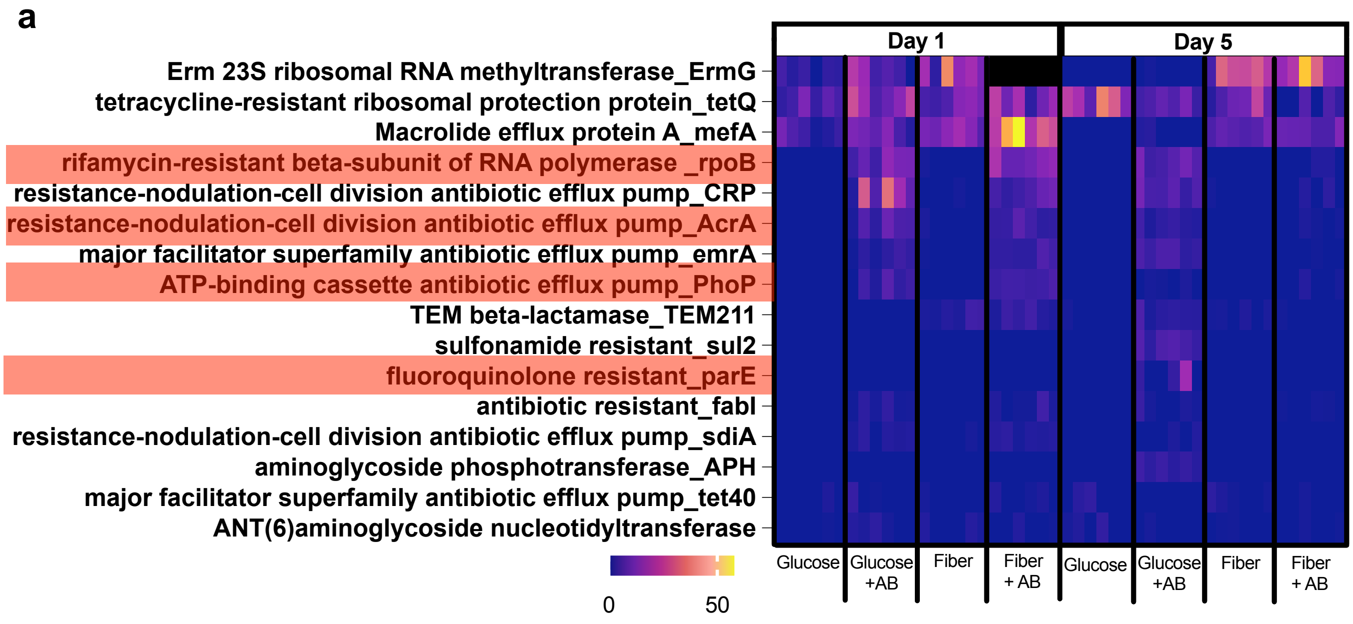

Supplementary Figure 4: (a) Antibiotic resistance gene (ARG) expression quantified using ShortBRED and Comprehensive Antibiotic Resistance Database (CARD) database from metatranscriptomic data. Proteobacteria unique ARGs highlighted in red. Black boxes exceed scale by 2x. Scale shows RPKM (reads per kilobase of transcript per million reads mapped).

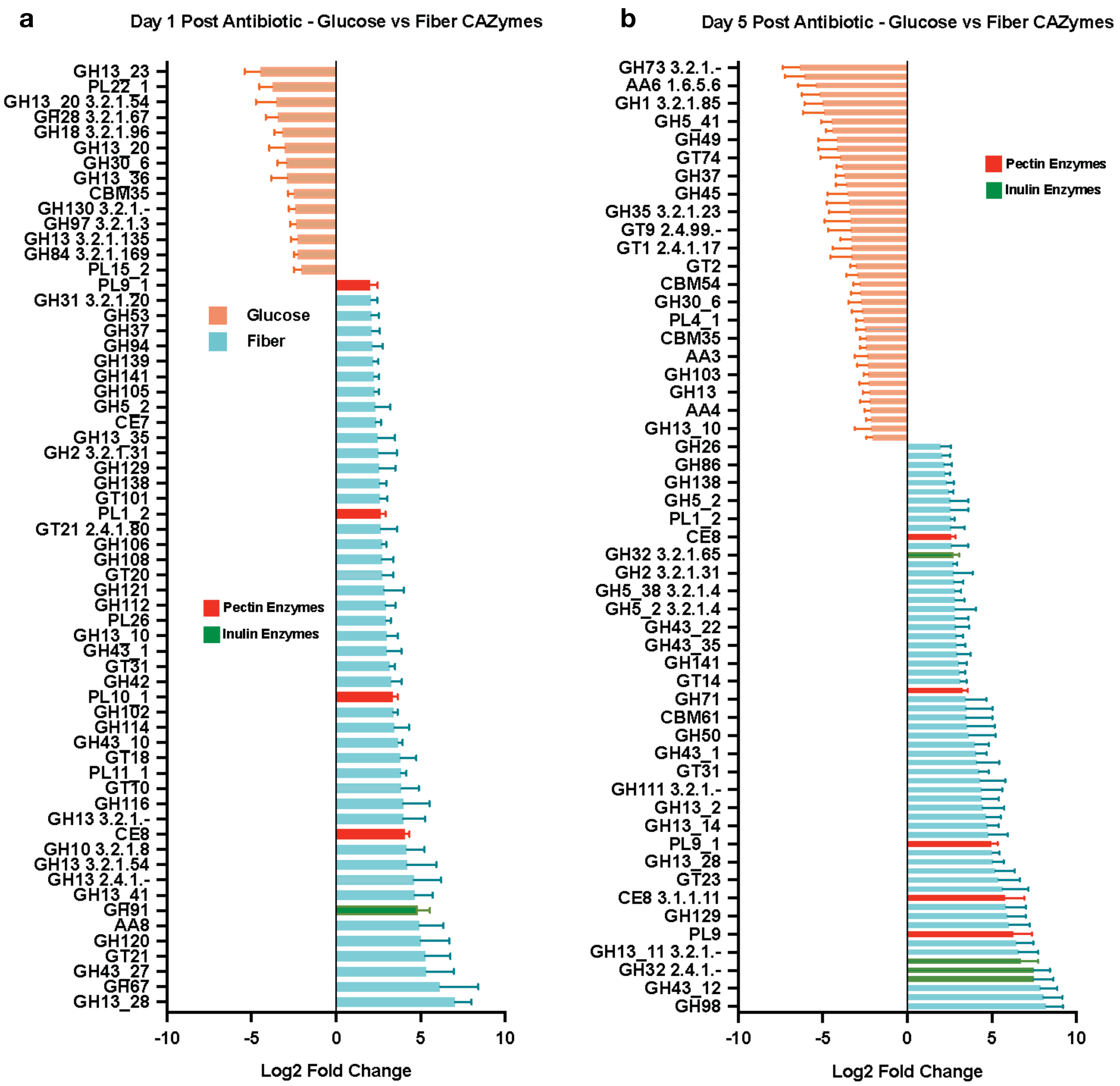

Supplementary Figure 5: DESeq2 analysis of short read metatranscriptomic data aligned to the CAZy database showing significant increases in Glucose + AB (orange) and Fiber + AB (blue). padj<0.05 and log2 FC >2 (n=6) Log2FC±SEM. CAZymes related to degradation of pectin shown in red and inulin shown in green.

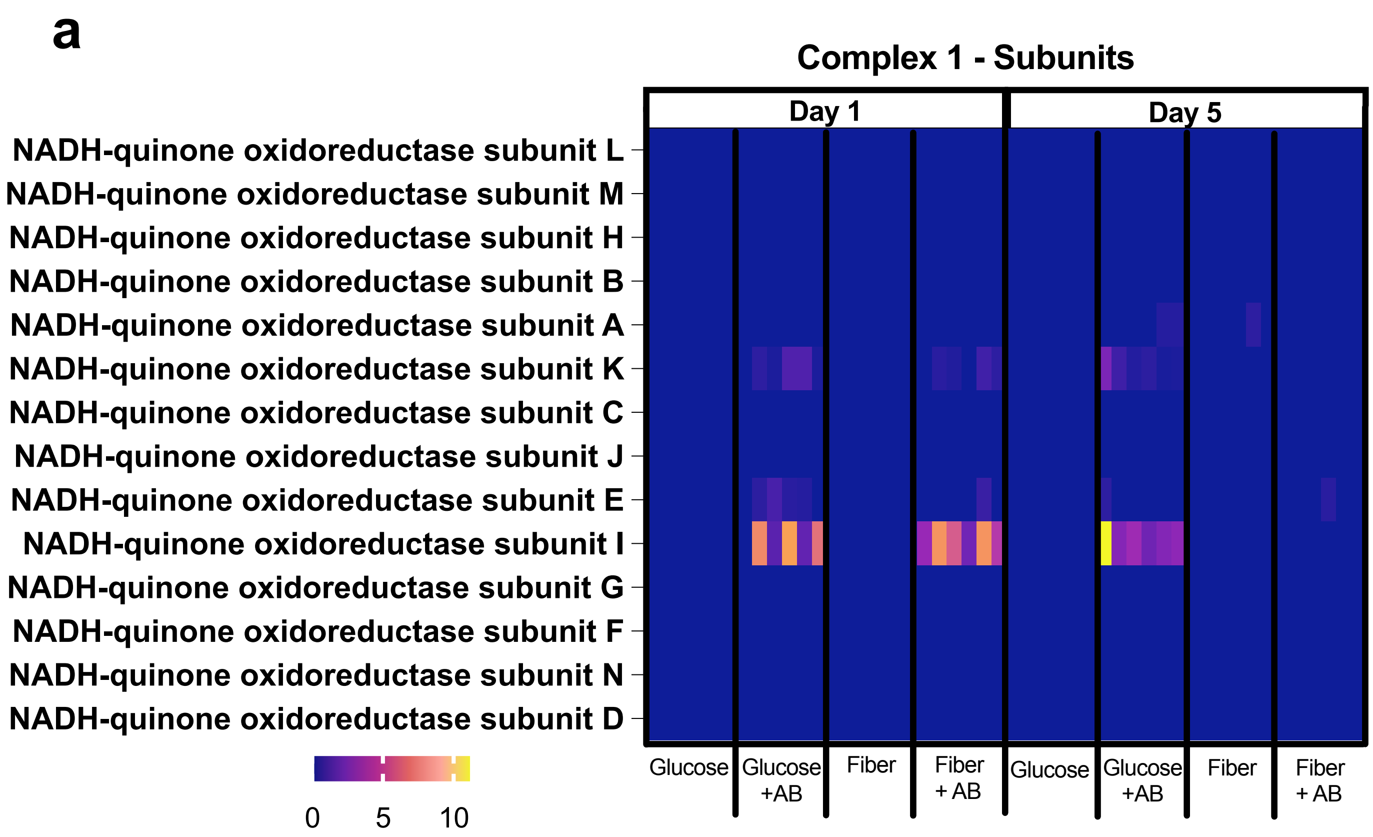

Supplementary Figure 6: (a) Complex 1 subunits quantified using ShortBRED from metatranscriptomic data. Scale shows RPKM (reads per kilobase of transcript per million reads mapped).

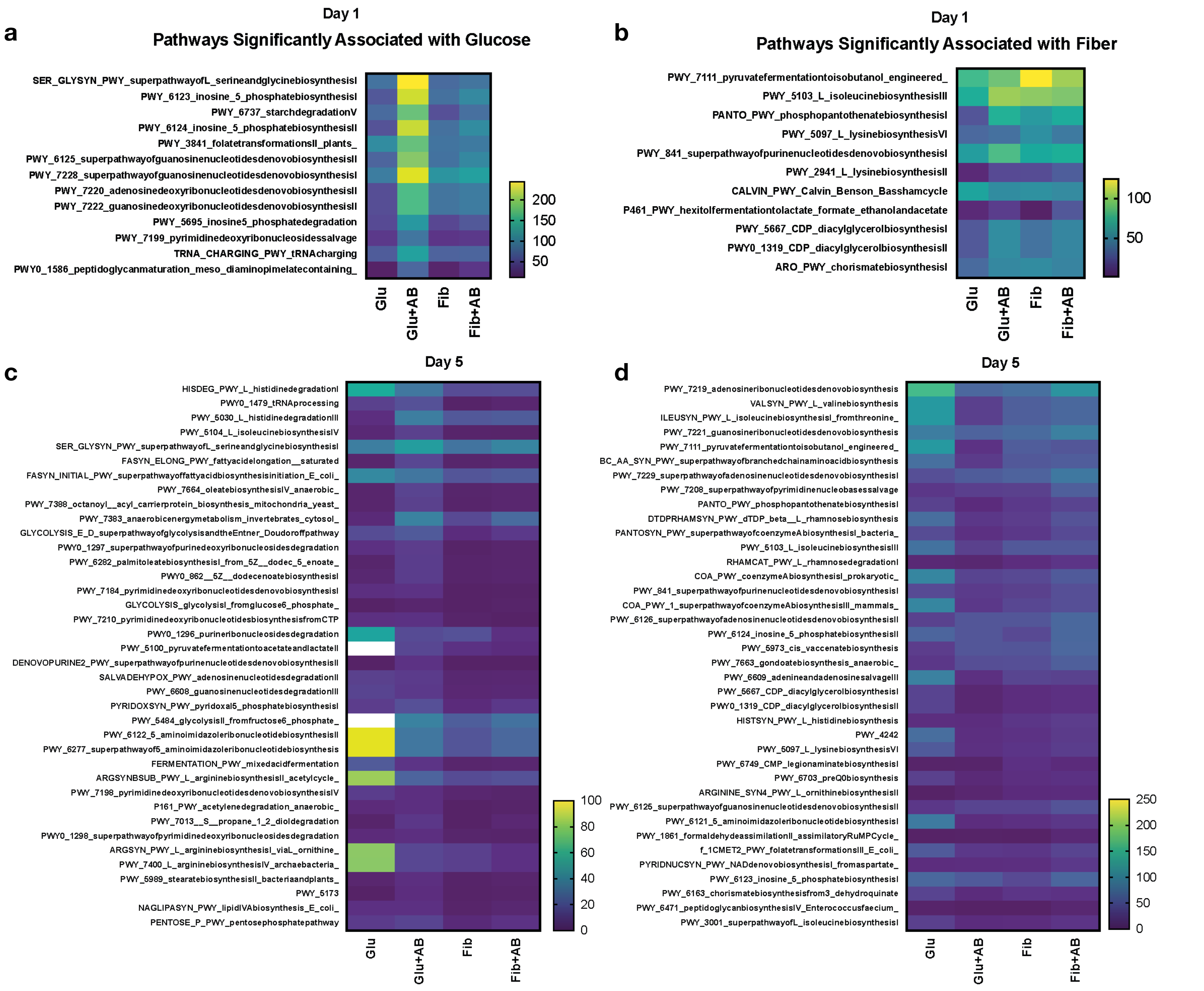

Supplementary Figure 7: Heat map of relative expression of HUMaN3 pathways (cpm) significantly associated on Day 1 of the experiment with Glucose+AB (A) and Fiber+AB (B) as determined by LDA (Linear Discriminant Analysis) using LEfSe. Cutoffs: LDA > 3 and p-value < 0.05. Significantly associated pathways on Day 5 of the experiment in Glucose+AB (C) and Fiber+AB (D) (n=6). Full pathway results in Supplementary Information.

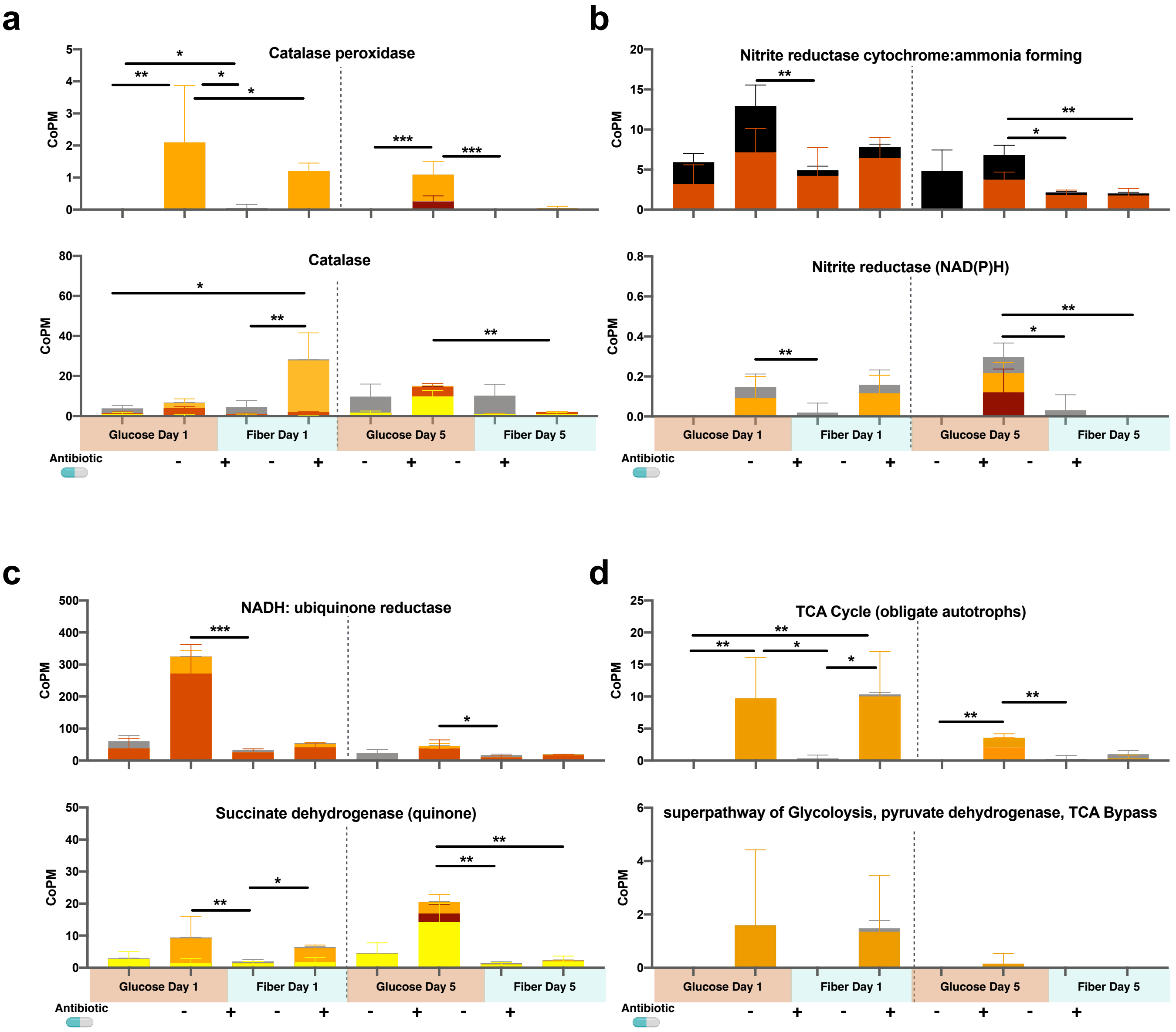

Supplementary Figure 8: HUMaN3 reaction expression during D1 and D5 of experiment. (a) Aerobic metabolism, top – Catalase peroxidase adj values left to right = 0.0049, 0.0138, 0.0138, 0.0360, 0.0009, 0.0009. bottom – Catalase adj p values left to right = 0.0042, 0.0057, 0.0017. (b) Nitrate metabolism, top – Nitrite reductase cytochrome adj p values left to right = 0.0076, 0.0115, 0.0065, bottom – Nitrite reductase NADPH adj p values left to right = 0.0076, 0.0115, 0.0065. (c) Oxidative phosphorylation top - NADH ubiquinone reductase adj p values left to right = 0.0006, 0.0374, bottom – Succinate dehydrogenase adj p values left to right = 0.0087, 0.0374, 0.0023, 0.0065.(d) TCA cycle, top – TCA cycle obligate autotrops adj p values left to right = 0.0041, 0.0041, 0.0495, 0.0495, bottom – TCA Bypass. For a-d: Copm = copies per million reads. Kruskal Wallis with Dunn's Correction (n=6) Mean±SEM. $*p < 0.05$; $**p < 0.01$; $***p < 0.001$; $****p<0.0001$.

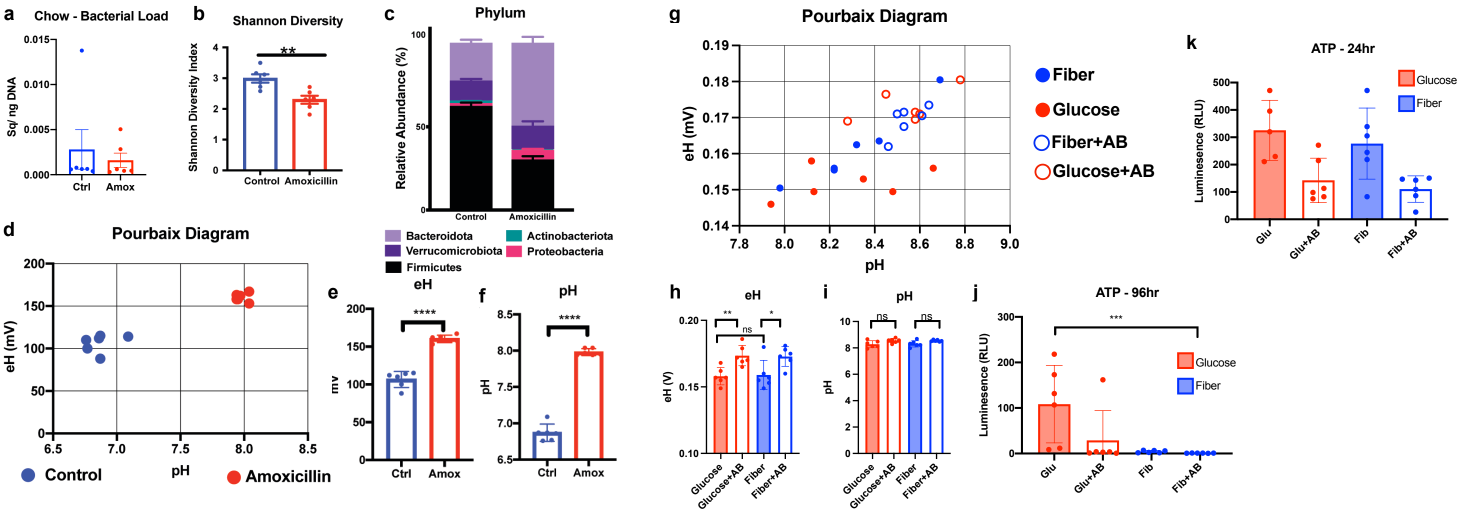

Supplementary Figure 9: (a) Bacterial load measured by qPCR from mice on the chow diet. (n=6) Mean±SEM No significance found with two-tailed unpaired t-test. (b) Shannon diversity values. (n=6) Mean±SEM p value = 0.0045 using two-tailed unpaired t-test. (c) Relative abundance of bacterial phyla. (n=6) Mean±SEM (d) Pourbaix diagram depicting eH and pH values from lyophilized cecal contents of mice on a chow diet with and without antibiotics measured within 24hrs of rehydration with RO water (n=6).(e) redox potential (eH) p value = <0.0001 with two-tailed unpaired t-test, (n=6) Mean±SEM (f) pH p value = <0.0001 with two-tailed unpaired t-test, (n=6) Mean±SEM (g) Pourbaix diagram depicting eH and pH values from lyophilized cecal contents of mice on a 20% glucose and 20% fiber diet with and without antibiotics measured within 96hrs of rehydration with RO water (n=6). (h) eH, p values left to right = 0.0043, 0.0455 (n=6) Mean±SEM Significance determined by two-tailed Mann-Whitney test and (i) pH (n=6) Mean±SEM No Significance determined by two-tailed Mann-Whitney test. (j) ATP levels measured from rehydrated cecal contents that were frozen and lyophilized immediately upon collection. Samples from Figure 4 h,i, (n=6) Mean±SEM, adj p value = 0.0003. Kruskal Wallis with Dunn's Correction (k) ATP levels measured from samples in Supplementary Figure 9 g,h,i (n=6)Mean±SEM Kruskal Wallis with Dunn's Correction ∗p < 0.05; ∗∗p < 0.01; ∗∗∗p < 0.001; ∗∗∗∗p < 0.0001.

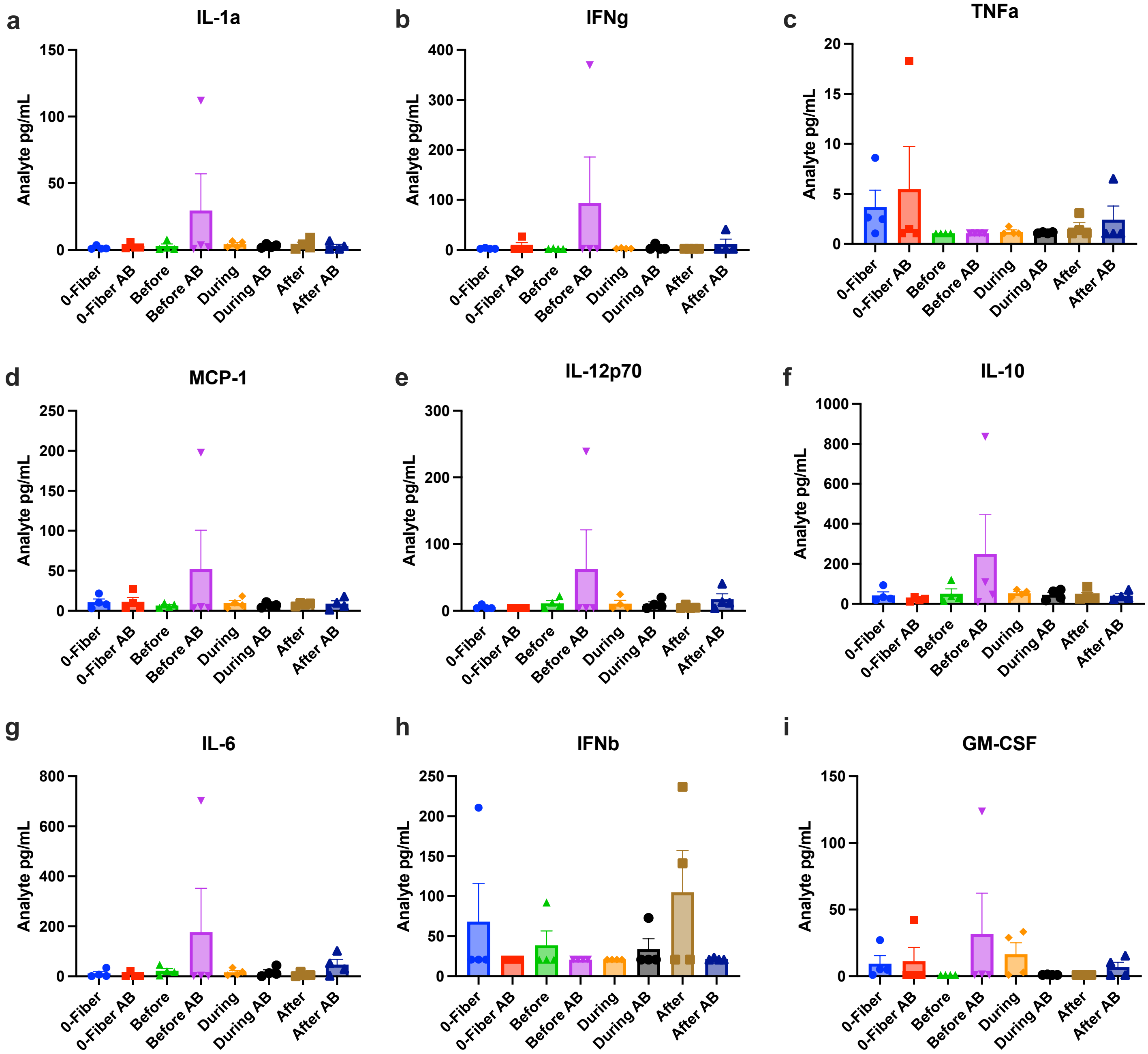

Supplementary Figure 10: Serum cytokine panel from mouse experiment depicted in Figure 1. (a-i) analyte shown pg/mL. (n=4 mice) for all cytokines assessed with 2 technical replicates per mouse. No significance found. Kruskal Wallis with Dunn's Correction.
