# Supplementary material for: Fiber supplementation protects from antibiotic-induced gut microbiome dysbiosis by modulating gut redox potential
Source: Nat Commun. 2023 Aug 24;14:5161. doi: 10.1038/s41467-023-40553-x (PMC10449846; doi:10.1038/s41467-023-40553-x)
Supplement: Supplementary file 3 — Description of Additional Supplementary Files [file 41467_2023_40553_MOESM3_ESM.docx]

**File Name: Supplementary Data 1**

Description: Histopathology scores from blinded analysis of colon and small intestine samples stained with H&E from Day 5 of the experiment with Glucose, Glucose+AB, Fiber, Fiber+AB. Colons from 6 mice assessed from each group. Small Intestines from 6 mice assessed from each group except the glucose group which contains 4 mice. All slides contain 2 tissue sections. Pathologist searched for markers of inflammation, hyperplasia, ulceration, villi length. No significant difference found by pathologist at CCHMC. Representative images shown in Supplementary Figure 2a.

**File Name: Supplementary Data 2**

Description: Complex 1 database construction information with Uniref90 cluster IDs. Sequences used to screen carbon-fixation pathways with accession numbers.

**File Name: Supplementary Data 3**

Description: DESeq2 (Differential gene expression analysis based on the negative binomial distribution) analysis of phylum and species level annotations from metagenomic short-read data with interaction.

**File Name: Supplementary Data 4**

Description: DESeq2 (Differential gene expression analysis based on the negative binomial distribution) analysis of REFSEQ organism annotation from metatranscriptomic data. Interaction and main comparisons shown.

**File Name: Supplementary Data 5**

Description: DESeq2 (Differential gene expression analysis based on the negative binomial distribution) analysis of REFSEQ function and SEED function annotation from metatranscriptomic data. Interaction and main comparisons shown.

**File Name: Supplementary Data 6**

Description: Significant Pathways identified from MaAsLin2 (Microbiome Multivariable Associations with Linear Models) from diet and diet+drug interaction.

**File Name: Supplementary Data 7**

Description: Extended pathway data from HUMAnN3 analysis of metatranscriptomic reads with LDA scores shown for comparison between all 4 groups. LEfSe (Linear discriminant analysis Effect Size)

**File Name: Supplementary Data 8**

Description: Supplementary data from redox potential plot in Figure 4a with abundances of MetaCyc pathways from metatranscriptomic reads at Day 5 of Experiment. Significant reactions shown from analysis with MaAsLin2 (Microbiome Multivariable Associations with Linear Models). MetaCyc annotation and deltaG of reactions shown.

**File Name: Source Data**

Description: Full PERMANOVA results with effect sizes, F values, p values from Figure 1e and Figure 2c.
